# Unexpected Transcellular Protein Crossover Occurs During Canonical DNA Transfection

**DOI:** 10.1002/jcb.24884

**Published:** 2014-10-15

**Authors:** Jason Arsenault, Sabine AG Cuijpers, Dhevahi Niranjan, Bazbek Davletov

**Affiliations:** 1 MRC-Laboratory of Molecular Biology, Neurobiology DivisionCambridge, CB2 0QH, UK; 2 Department of Pharmaceutical Sciences, University of TorontoToronto, Canada, M5S 3M2; 3 Department of Biomedical Sciences, University of SheffieldSheffield, S10 2TN, UK

**Keywords:** TRANSFECTION, LIPOFECTION, BOTULINUM NEUROTOXIN, ENDOCYTOSIS, TRAFFICKING, INTERNALIZATION, SNARE

## Abstract

Transfection of DNA has been invaluable for biological sciences, yet the effects upon membrane homeostasis are far from negligible. Here, we demonstrate that Neuro2A cells transfected using Lipofectamine LTX with the fluorescently coupled Botulinum serotype A holoenzyme (EGFP-LcA) cDNA express this SNAP25 protease that can, once translated, escape the transfected host cytosol and become endocytosed into untransfected cells, without its innate binding and translocation domains. Fluorescent readouts revealed moderate transfection rates (30–50%) while immunoblotting revealed a surprisingly total enzymatic cleavage of SNAP25; the transgenic protein acted beyond the confines of its host cell. Using intracellular dyes, no important cytotoxic effects were observed from reagent treatment alone, which excluded the possibility of membrane ruptures, though noticeably, intracellular acidic organelles were redistributed towards the plasma membrane. This drastic, yet frequently unobserved, change in protein permeability and endosomal trafficking following reagent treatment highlights important concerns for all studies using transient transfection. J. Cell. Biochem. 115: 2047–2054, 2014. © 2014 Wiley Periodicals, Inc.

The delivery of exogenous proteins into cells and tissues has been invaluable for biological sciences and medicine [Gao and Huang, 1995]. These modulations of intracellular parameters brought forth by the addition of heterologous genes, following the advent of cloning methodologies, greatly accelerated discovery platforms in all biochemical fields [Geisse and Voedisch, 2012].

Transfection, a non-viral mediated genetic transfer, relies on a number of different yet consequently similar approaches. Reagents such as polycationic liposomes and polymers form cargo bound liposomes known as lipoplexes or polyplexes respectively [Tros de Ilarduya et al., 2010] that bind to extracellular membranes to be internalized. Other chemical reagents such as calcium phosphate or cyclodextrins and physical treatments such as electroporation and sonoporation can also promote the intracellular delivery of the genetic material [Graham and van der Eb, 1973; Neumann et al., 1982; Song et al., 2007; Menuel et al., 2008]. Alternatively, biolistic delivery mechanisms can directly fire DNA-coated nanoparticles into cells and tissues for localized exogene expression [O'Brien and Lummis, 2011; Arsenault and O'Brien, 2013].

The most commonly used approaches however, utilize polycationic lipids and polymers [Kim and Eberwine, 2010]; a wide range of these different formulations are commercially available [Caracciolo and Amenitsch, 2012]. Taking advantage of the overall anionic charge on the extracellular surface of the plasma membranes, these lipoplexes bind onto, and get internalized into the cells through endocytosis. Following a yet not fully understood mechanism, the DNA cargo gets released where it can subsequently be transcribed in the nucleus [Tarahovsky et al., 2004; Caracciolo and Amenitsch, 2012; Liu and Zhang, 2012; Lonez et al., 2012]. Current advances are aimed towards improving the yields of transfections and lowering the cellular toxicity [Liu and Zhang, 2012; Lonez et al., 2012; Nguyen and Szoka, 2012]. These formulations inherently interact with and modulate the plasma membrane's ability to internalize the complexed cargos [Caracciolo and Amenitsch, 2012; Lonez et al., 2012]. These same types of reagents have also been exploited to deliver protein cargos themselves into cells [Zelphati et al., 2001; Oba and Tanaka, 2012]. Yet there is limited data on the ability of the translated exogenous protein, if released from the primary host cell, to subsequently penetrate into reagent treated but non-transfected cells.

In order to determine transfection yield, researchers often rely on fluorescently tagged chimeras, immunostaining, and radioligand binding (or radio-labeling) [Bajohrs et al., 2004; Ramos-Vara, 2005; Arsenault et al., 2010a,2010b]. These methods, nevertheless having their own innate advantages, might not have the necessary sensitivity to detect very low levels of the exogenous protein.

Here, we show that a SNAP25 protease [Jahn et al., 2003; Montal, 2010; Davletov et al., 2012], the Botulinum neurotoxin serotype 1A (BoNT/A) holoenzyme (LcA), lacking its innate cell surface binding and translocation domains, tagged with enhanced green fluorescence protein (EGFP), once expressed inside an initial host cell can escape, at undetectable concentrations, and subsequently be internalized into a secondary, reagent treated, yet non-transfected cell. Since these proteases, which are potent exocytotic inhibitors, have previously been shown permeable into cells treated with transfection reagents, such as Fugene HD, Lipofectamine 2000, and Lipofectamine LTX [Kuo et al., 2010; Arsenault et al., 2014], it is surprising that their ability to migrate from cell-to-cell, following transient expression, has never been addressed. In fact, there is very little investigation as to whether any protein can re-enter secondary cells once transiently expressed even though they might have been shown amenable to lipid mediated delivery themselves. These observations become extremely problematic in transfection studies where highly potent proteins are investigated (e.g., transcription factors, enzymes, and toxins) [Montagne et al., 2012; Arsenault et al., 2013], in cases where cells are compared to their neighbors based on visual confirmation of the exogenous protein, when the transfected cells are separated and independently analyzed, when transfected proteins are used as a readout to follow trafficking processes.

To investigate whether these observation were restricted to the BoNT/A Lc we also verified the BoNT/E holoenzyme; the results were identical. We also determined that the Ricin holoenzyme shows an over 200-fold increase in efficacy in cells treated with the same transfection reagent; this untargeted enzyme can thus also penetrate transfected cells indiscriminately [Lord and Spooner, 2011; Arsenault et al., 2014]. FITC-conjugated peptides could be detected within organelles of transfected but not untransfected cells. These results reveal an altered membrane homeostasis and binding capacity as well as altered endosomal trafficking following lipofection. These results should be strongly considered for proper conclusive interpretations of any transfection study.

## MATERIALS AND METHODS

Full materials and method has been included as supplementary information. Briefly, cell culturing, immunocytochemistry, cytotoxicity assays, western immunoblotting, flow cytometry, and confocal microscopy was performed as described elsewhere [Arsenault et al., 2014]. Transfection of Neuro2A cells was performed as manufacturer recommends using pDNA vectors described elsewhere [Bajohrs et al., 2004]. Peptides and proteins used in this study were described previously [Arsenault et al., 2013; Arsenault et al., 2014]. Annexin V-FITC and Ricin A chain were obtained from Sigma-Aldrich (Dorset, UK).

## RESULTS

### DISCREPANCIES IN TRANSFECTION YIELDS

**Fig 1:**
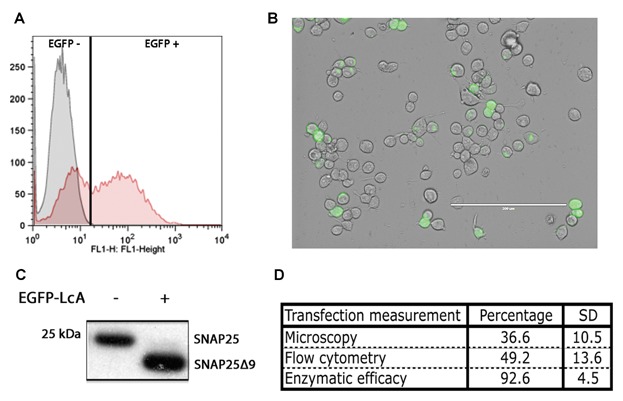
Measuring transfection yields. A: Flow cytometry analysis of Mock (gray) and EGFP-LcA (red) transfected Neuro2A cell distribution shown as a histogram of fluorescent intensity (FL1). B: Confocal microscopy image of EGFP-LcA transfected Neuro2A cells. White bar: 200 μm. C: Western immunoblotting of total SNAP25 (SMI81) showing the enzymatic activity of EGFP-LcA on transfected Neuro2A cells. A total conversion of SNAP25 can be observed. D: Calculated transfection percentage using the above three interpretation methodologies. There is a strong discrepancy between the fluorescently observed tranfection yields compared to total intracellular enzymatic activity.

Following transient transfection, various methods can be used to determine the relative exogene expression yields. As can be seen in Figure 1, various traditional methods to quantify the expression of the transient exogene EGFP-LcA expression using Lipofectamine LTX into Neuro2A cells are presented. Using flow cytometry cells were gated by their forward and side-scatter (size and complexity) to select only morphologically normal cells. These gated cells were then quantified proportional to their EGFP fluorescent intensity (Fig. 1A). We also verified EGFP expression using confocal microscopy (Fig. 1B). Cells expressing the EGFP-LcA present a fluorescence that is generally localized around the cytosolic face of the plasma membrane. Western immunoblotting was also used to determine the total enzymatic effect within the entire sample. Total SNAP25 was immunoblotted using the SMI81 antibody revealing both the intact and the BoNT/A cleaved fragment. As can be seen in Figure 1C, the cleavage of the 9 C-terminal residues (SNAP25Δ9) leads to the appearance of a lower MW band. Surprisingly, this readout shows an improbably high transfection percentage. Figure 1D shows these quantifications where both flow cytometry and confocal microscopy correlates well despite the extra-ordinarily high enzymatic efficacy.

### ALTERNATIVE MODES OF PROTEASE ENTRY AND SPREAD OF THE EXOGENOUS PROTEIN BEYOND THE INITIAL HOST CELL

**Fig 2:**
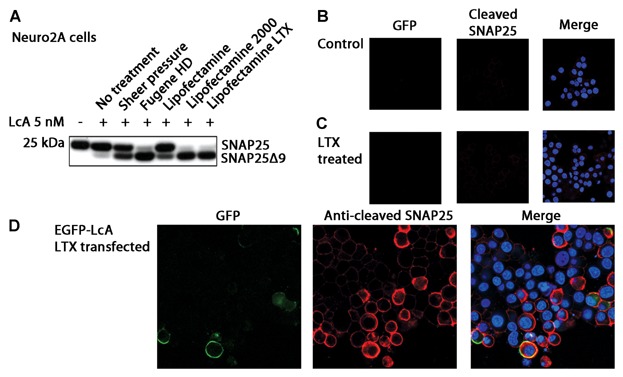
Various modes of cellular entry of the type A protease (LcA) and immunoblotting of cleaved SNAP25. A: Western immunoblotting of total SNAP25 (SMI81) for Neuro2A cells treated with the type A protease under various conditions. Stressors such as sheering force or transfection reagents applied to the cells can mediate the entry of the protease. B–D: Confocal microscopy of cells immunostained with anti-cleaved SNAP25 antibody (red) and Hoechst stain (blue): (B) Control cells, (C) Mock-transfected cells, and (D) EGFP-LcA transfected cells (green). The anti-cleaved SNAP25 immunoreactivity can only be seen in cell populations subjected to LcA; this signal also extends beyond the detectable expression of the EGFP fluorophore.

As was previously shown [Kuo et al., 2010; Arsenault et al., 2014], transfection reagents can mediate the intracellular entry of BoNT holoenzymes. Figure 2A shows different conditions where unintended protease penetration can occur. Sheer force, representative of physical transfection methods, and different transfection reagents can cause the “transduction“ of the protease.

To determine how intimately the fluorescent signals and protease activity correlates, we performed immunocytochemistry using a highly specific BoNT/A cleaved SNAP25 antibody. As can be seen in Figure 2B,C, there is no background EGFP nor cleaved SNAP25 signal in both the untreated and mock transfected Neuro2A cells. However, when EGFP-LcA is expressed within Neuro2A cells we observe a membrane localized EGFP signal as was previously shown in Figure 1B and was previously reported elsewhere [Foran et al., 2003; Bajohrs et al., 2004; Arsenault et al., 2014]. Nevertheless, we also observe a much more promiscuous anti-cleaved SNAP25 immunoreactivity beyond where lies the EGFP signal. This observation corroborates the transfection yield discrepancies.

### RELEASE OF SNARE PROTEASE FROM HOST CELL AND TRANSFECTION REAGENT MEDIATED RE-ENTRY

**Fig 3:**
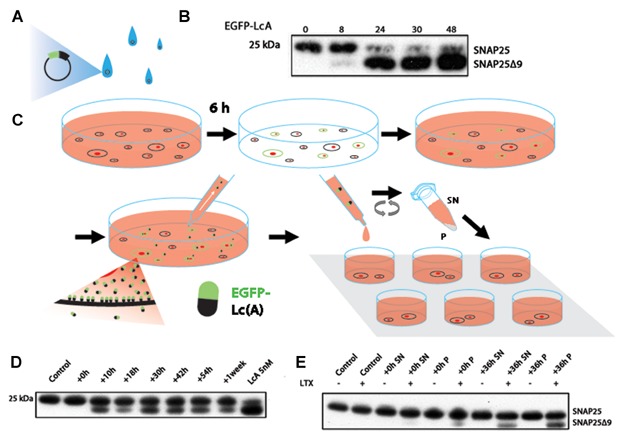
Analyzing the EGFP-LcA transcellular protein crossover by monitoring enzymatic activity. A: Neuro2A cells were transfected with EGFP-LcA; 6 h after transfection the medium was discarded, cells were washed with PBS, and new complete DMEM was added. B: Western immunoblotting of SNAP25 showing the total enzymatic efficacy of EGFP-LcA at different time points (h). C: The new medium was sampled at intervals. This sampled medium, absent of exogenous DNA, was applied to fresh Neuro2A cells treated with LTX then incubated for 42 h. This medium sampled from previously transfected cells was then centrifuged to separate soluble (supernatant, SN) and suspended (pellet, P) fractions. These fractions were added onto new Neuro2A cells treated with or without Lipofectamine LTX. D: Western immunoblotting of SNAP25 shows enzymatic efficacy within the untransfected Neuro2A cells where the sampled medium was applied and treated with Lipofectamine LTX. The previously sampled medium thus contains the EGFP-LcA protein as detected by enzymatic activity. E: Western immunoblotting of SNAP25 shows that soluble (SN) and suspended membrane bound (P) EGFP-LcA are found freely floating in the medium and can, mediated by Lipofectamine LTX, enter new Neuro2A cells to cleave intracellular SNAP25.

To ascertain whether a much higher percentage of transfection actually occurs but lies below the threshold of detection or whether the EGFP-LcA can escape the confines of these transfected cells to cleave SNAP25 in non-transfected cells we sampled the culture medium at different time points. Figure 3 shows a schematic of this transfection protocol. Figures 3A and B shows the total enzymatic readout within undisturbed EGFP-LcA transfected Neuro2A cells; after 24 h a substantial amount of SNAP25 cleavage can already be observed. This culture medium, which was replaced 6 h after transfection, was then sampled at different time points and administered to new Neuro2A cells that were treated with Lipofectamine LTX only (Fig. 3C). Since the cells were washed 6 h after transfection, we removed the remaining free-floating DNA-lipoplexes that could have been carried over to the second generation of cells. Figure 3D shows that the protease-contaminated culture medium drawn from the first generation of transfected cells as early as 10 h post-wash (16 h post-transfection) can cleave SNAP25 in a second generation of cells. To avoid seeding the wells with pre-cleaved SNAP25 we performed western immunoblotting on the supernatant alone which showed no immunoreactive SNAP25 nor SNAP25Δ9 while resuspended EGFP-LcA transfected cells clearly did (Supplementary Fig. S1). Thus we were not inadvertently transferring EGFP-LcA transfected cells into new wells. Also, as the 0 h post-wash seeding does not show any cleaved-SNAP25 in the second generation of Neuro2A, meaning that the primary cells require synthesis time, this excludes the possibility of inadvertent DNA-Lipoplex carry over. To test this further, we centrifuged the sampled medium (Fig. 3C). As can be seen in Figure 3E, both the soluble (supernatant, SN) and the suspended (pellet, P) fractions contained the protease. And this also shows that the penetration of the proteases is dependent on the presence of the transfection reagent, in this case Lipofectamine LTX. Thus, once the soluble or suspended proteases are released into the culture medium, they can, mediated by the transfection reagent, enter new untransfected cells where they continue their innate functions.

### ABSENCE OF CYTOTOXICITY BUT OBSERVABLE REDISTRIBUTION OF ACIDIC ORGANELLES

**Fig 4:**
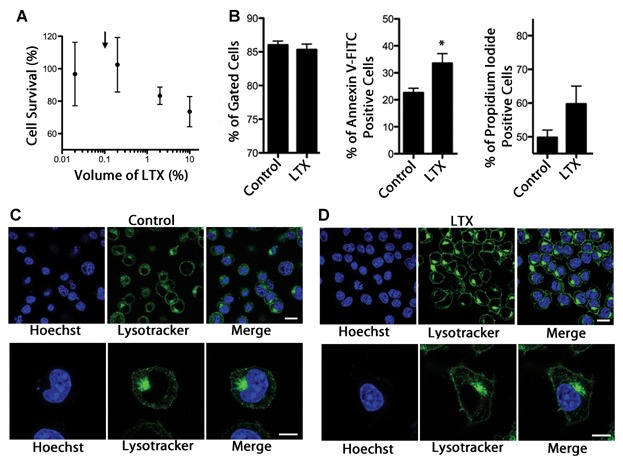
Investigating Lipofectamine LTX effects on neuro2A cells. A: CCK-8 assay on Neuro2A cells treated with various doses of Lipofectamine LTX. The consistently used experimental dose is indicated by an arrow. B: Flow cytometry assays show the percentage of morphologically normal cells (left panel), the amount of Annexin V-FITC labeling (middle), and propidium iodide labeling (right). C,D: Live imaging of Neuro2A cells treated with or without Lipofectamine LTX and Lysotracker green. C: Normal distribution of acidic organelles in untreated Neuro2A cells at low magnification (top) and high (bottom). D: Altered distribution and intensity of acidic organelles following Lipofectamine LTX treatment shown at low magnification (top) and high (bottom). Lysotracker green strongly labels the area surrounding the plasma membrane. Low magnification white bar: 20 μm. Higher magnification white bar: 10 μm.

To ascertain whether lipofectamine LTX could adversely affect Neuro2A cells we performed various survival assays. CCK-8 assay can quickly and efficiently report any cell deaths by monitoring the colorimetric transition of a tetrazolium salt proportional to the amount of viable cells. As can be seen in Figure 4A, the concentration of lipofectamine LTX used (black arrow) did not display any significant loss of viability in Neuro2A cells. Figure 4B shows three readouts obtained from flow cytometry. The left panel shows the total proportion of cells bearing a normal morphology as determined by forward and side scatter. There was no significant disruption in the size and complexity of Neuro2A cells treated with lipofectamine LTX compared to untreated controls. However, a moderate elevation was observed in Annexin V-FITC labeling compared to control (*P* < 0.05; middle panel) and a slight but non-significant elevation in propidium iodide labeling. If the cytoplasmic membranes were perforated by the reagent there should have been a substantial elevation in propidium iodide labeling. These effects were nevertheless very subtle. As a previous study had shown that the use of bafilomycin A, an endosomal protein pump inhibitor, could prevent the lipofection mediated entry of Botulinum neurotoxins [Kuo et al., 2010] we used lysotracker green to investigate the distribution of acidic organelles in Neuro2A cells. Firstly, there seemed to be a larger overall increase in the total fluorescent signal or total fluorophore uptake in Neuro2A cells that were treated with lipofectamine LTX. Furthermore, the distribution of acidic endosomal compartments were strongly distributed around the plasma membrane indicating an active endocytotic/exocytotic process. Evidently, this active cellular trafficking would undoubtedly contribute to cargo endocytosis and concomitant exocytosis. Unfortunately, due to proprietary concerns we were unable to investigate the individual contribution of the lipofectamine LTX ingredients.

**Fig 5:**
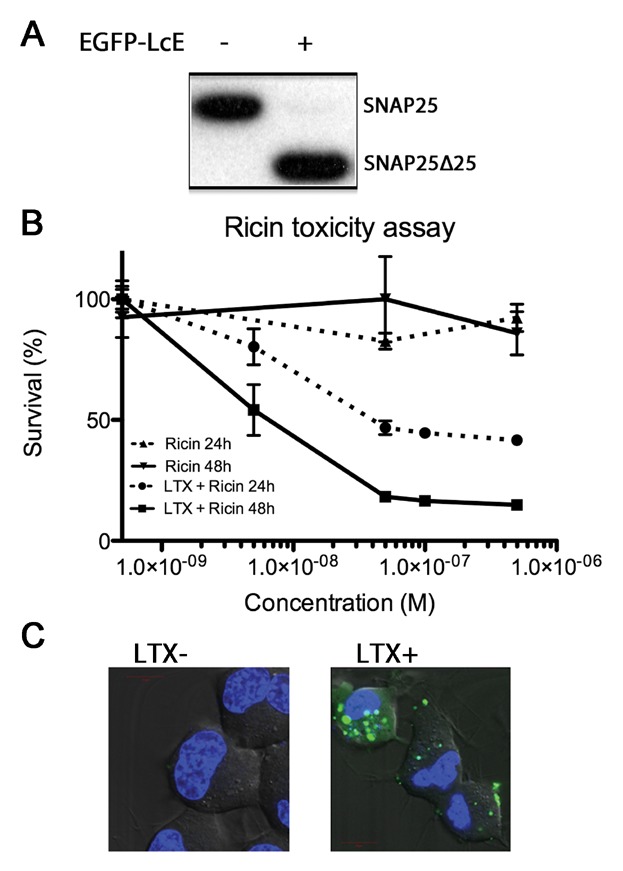
Other proteins having heightened efficacies in presence of Lipofectamine LTX. A: Western immunoblotting of Neuro2A cells transfected with EGFP-LcE also shows a complete cleavage of SNAP25. B: Cell survival assay using the enzymatic subunit of Ricin. Cells previously treated with LTX show an over 200-fold gain in sensitivity towards the untargeted holoenzyme. C: FITC-Syntaxin (201–245) was applied to Neuro2A with (right panel) or without (left panel) Lipofectamine LTX. Only cells treated with Lipofectamine LTX were able to internalize the fluorescent peptide to detectable levels.

Whether the enhanced protease internalization observed might be a peculiarity of the membrane localization capacity of the BoNT/A holoenzyme [Fernandez-Salas et al., 2004], we performed the same tests with the type E protease (EGFP-LcE) a non-membrane bound SNAP25 protease [Foran et al., 2003]. As can be seen in Figure 5A, western immunoblotting of total SNAP25 also reveals a near total SNAP25 cleavage while confocal microscopy and flow cytometry only detected EGFP-LcA type expression patterns (approximately 40%; data not shown). We also undertook cell survival assays of Neuro2A cells treated with the ricin holoenzyme, a ribosomal inactivating protein, in the presence or absence of Lipofectamine LTX. As can be seen in Figure 5B, there is no detectable cell death 24 and 48 h after this untargeted toxin is incubated with the cells. However, with cells pre-treated with lipofectamine LTX, a substantial amount of cell death can be observed at 24 h and, increasingly so, at 48 h post administration. Also, and as was previously determined [Arsenault et al., 2014], peptides can be internalized via transfection reagents. Figure 5C shows Neuro2A cells treated with or without Lipofectamine LTX and 10 μg/ml of FITC-Syntaxin (201–245) was added to the culture medium for 30 min. Following, this incubation cells were washed in PBS and the medium was replaced. As can be seen, a noticeable amount of endosomal fluorescence is detectable in the LTX treated Neuro2A cells but not without pre-treatment. These results show that other proteins and peptides display a similar ability to be efficiently internalized by Lipofectamine LTX treated cells.

## DISCUSSION

Since, a great number of laboratories consistently rely on transient transfection to tackle important biological and medical questions, it is crucially important to properly address the ability of the exogenous protein to act beyond the confines of its intended cellular target. Lipofectamine LTX, as well as other transfection reagents, is currently under proprietary control, which precludes the analysis of its individual constituents to determine which moiety facilitates these entries. As was previously determined with BoNT/A and E, the use of bafilomycin A1, an endosomal H^+^ pump inhibitor, could prevent protein transduction, indicating that endocytosis followed by translocation rather than membrane perforation was the contributing cause [Kuo et al., 2010]. Our data also supports this concept as membrane perforation would have affected both propidium iodide and CCK-8 results, thus the entry mechanisms are not due to exorbitant membrane perforations or breakage resulting from necrosis or late apoptosis. Although there was a slight yet significant elevation in the level of Annexin V-FITC binding, this could also be a false positive as, like the BoNT/A holoenzyme that localizes on the cytosolic face of the plasma membrane [Bajohrs et al., 2004; Fernandez-Salas et al., 2004; Wang et al., 2011], this protein has membrane binding properties which could evidently be affected by this membrane homeostasis disruption. This would also correlate with other reports which indicated that Lipofectamine LTX delivered DNA via the clathrin-mediated endocytosis pathway, a mechanism traditionally relied upon by Botulinum molecules, although these transgenes lacked the translocation domain required for endosomal escape [Wong et al., 2007; Caracciolo et al., 2010]. These observations elicit important questions related to the luminal to cytosolic translocation of Botulinum neurotoxins.

Another advantage of using BoNTs is that they inhibit exocytosis. This strongly suggests that the exit pathways are not driven by SNARE mediated exocytosis. Since BoNT/A and BoNT/E can significantly inhibit exocytosis and both still show almost total SNAP25 cleavage with transfection rates of less than 50%, it would be unlikely that exocytosis is the driving force for cellular escape. As only a few molecules are necessary within the cytosol to substantially cleave SNAP25, the cells that do express these heterologous proteins, as observed by fluorescently-tagged readout, far exceed the requirements to completely cleave SNAP25 within their host cytosol. That being said, if those cells were to die, their intracellular contents would be spilled into the culture medium and thus able to cross over into neighboring untransfected cells. It is also important to note that both type A and E proteases display no discernible cytotoxicity in Neuro2A cells [Foran et al., 2003; Arsenault et al., 2014]. We also cannot exclude reverse translocation from the cytosolic compartment towards the extra-cellular medium or into exocytotic vesicle. As some peptides and proteins can enter cells either by pH dependent transcytosis through the endosomes or directly through the plasma membrane, little precludes their reverse transcytosis [Kuo et al., 2011; Madani et al., 2011; Liu and Zhang, 2012; Arsenault et al., 2014] if membrane polarities were disrupted. The contemporary readout methodologies shown in this study were not sensitive enough to address the escape issue, yet further research should be undertaken since all intracellular proteins could display escape kinetics following reagent treatment. Evidently, mock conditions are always necessary for any transfection study yet since they cannot address the effect of the transgene without transfection reagent, any conclusions taken from the experiment should consider the implications of transcellular effects.

These results do not necessarily abrogate future chemical transfection studies, but rather warns that increased amounts of controls should be undertaken depending on nature of the analysis. For example, expressing recombinant proteins for purification or targeting all cells for transformation might have little consequences, while however, comparisons of co-treated fluorescent and non-fluorescent cells might lead to profoundly erroneous interpretations. Furthermore, these observations might also be beneficial in co-transfection studies where multiple exogenous proteins must be present within the same cell [Liu et al., 2007]; the exogenous protein could then diffuse into neighboring cells missing the DNA coding for one of the cognate components. Miscalculations might occur when transfection percentage is corroborated to total functional efficacies. As we observe changes in endosomal trafficking following Lipofectamine treatment, studies that measure internalization kinetics following transient transfection should also be cautious of over interpretation as the transfection reagent itself might have potentiated and altered the endocytic/exocytotic balance and membrane homeostasis [Doherty and McMahon, 2008].

When studies rely upon canonical methods to detect the presence or absence of the exogenous protein, one should always inquire whether these methods are sufficiently sensitive to detect functionally relevant intracellular concentrations. Since, the visual reconnaissance threshold might readily surpass critical intracellular or intracompartmental concentrations at which the introduced protein could substantially affect cellular functions, overexpression evidently can aberrantly affect functionality [Cirigliano et al., 2013]. These questions would always depend upon those proteins' facility to exit and enter new cells and organelles as well as their innate potencies and functions within those locations. One way to circumvent this issue would be to utilize stable cell lines where, over a few generations of cellular division, the transfection reagents can be diluted out or degraded over time.

Although, we have observed that the BoNT/A holoenzyme can re-enter cells treated with Lipofectamine LTX, we have also shown that this internalization is not restricted to Botulinum proteases. Since proteins and peptides can be up-taken by cells, increased research should be undertaken in order to more properly define the interactions between diverse cell membranes, various proteins, and transfection reagents especially since the exact mechanisms of internalization or escape still are not fully understood [Madani et al., 2011; Caracciolo and Amenitsch, 2012; Lonez et al., 2012]. We have studied exogenous proteases and an N-glycosidase as well as endogenous protein fragments and they consistently showed an enhanced cellular internalization following reagent treatment. Evidently, there are numerous biotechnological and medical implications for such surprising membrane permeability. The delivery of SNARE protease, as well as other proteins and peptides, through the use of reagents, opens new doors for localized pharmaceutical delivery in basic research, biotechnology, and medicine.

## References

[b1] Antonucci F, Rossi C, Gianfranceschi L, Rossetto O, Caleo M (2008). Long-distance retrograde effects of botulinum neurotoxin A. J Neurosci.

[b2] Arsenault J, Cabana J, Fillion D, Leduc R, Guillemette G, Lavigne P, Escher E (2010a). Temperature dependent photolabeling of the human angiotensin II type 1 receptor reveals insights into its conformational landscape and its activation mechanism. Biochem Pharmacol.

[b3] Arsenault J, Cuijpers SA, Ferrari E, Niranjan D, Rust A, Leese C, O'Brien JA, Binz T, Davletov B (2014). Botulinum protease-cleaved SNARE fragments induce cytotoxicity in neuroblastoma cells. J Neurochem.

[b4] Arsenault J, Ferrari E, Niranjan D, Cuijpers SA, Gu C, Vallis Y, O'Brien J, Davletov B (2013). Stapling of the botulinum type A protease to growth factors and neuropeptides allows selective targeting of neuroendocrine cells. J Neurochem.

[b5] Arsenault J, Lehoux J, Lanthier L, Cabana J, Guillemette G, Lavigne P, Leduc R, Escher E (2010b). A single-nucleotide polymorphism of alanine to threonine at position 163 of the human angiotensin II type 1 receptor impairs Losartan affinity. Pharmacogenet Genomics.

[b6] Arsenault J, O'Brien JA (2013). Optimized heterologous transfection of viable adult organotypic brain slices using an enhanced gene gun. BMC Res Notes.

[b7] Bajohrs M, Rickman C, Binz T, Davletov B (2004). A molecular basis underlying differences in the toxicity of botulinum serotypes A and E. EMBO Rep.

[b8] Caracciolo G, Amenitsch H (2012). Cationic liposome/DNA complexes: From structure to interactions with cellular membranes. Eur Biophys J.

[b9] Caracciolo G, Callipo L, De Sanctis SC, Cavaliere C, Pozzi D, Lagana A (2010). Surface adsorption of protein corona controls the cell internalization mechanism of DC-Chol-DOPE/DNA lipoplexes in serum. Biochim Biophys Acta.

[b10] Cirigliano SM, Mauro LV, Grossoni VC, Colombo LL, Diament MJ, Kazanietz MG, Bal de Kier Joffe ED, Puricelli LI, Urtreger AJ (2013). Modulation of pancreatic tumor potential by overexpression of protein kinase C beta1. Pancreas.

[b11] Darios F, Niranjan D, Ferrari E, Zhang F, Soloviev M, Rummel A, Bigalke H, Suckling J, Ushkaryov Y, Naumenko N, Shakirzyanova A, Giniatullin R, Maywood E, Hastings M, Binz T, Davletov B (2010). SNARE tagging allows stepwise assembly of a multimodular medicinal toxin. Proc Natl Acad Sci USA.

[b12] Davletov B, Ferrari E, Ushkaryov Y (2012). Presynaptic neurotoxins: An expanding array of natural and modified molecules. Cell Calcium.

[b13] Doherty GJ, McMahon HT (2008). Mediation, modulation, and consequences of membrane-cytoskeleton interactions. Annu Rev Biophys.

[b14] Ekong TA, Feavers IM, Sesardic D (1997). Recombinant SNAP-25 is an effective substrate for Clostridium botulinum type A toxin endopeptidase activity in vitro. Microbiology.

[b15] Fernandez-Salas E, Steward LE, Ho H, Garay PE, Sun SW, Gilmore MA, Ordas JV, Wang J, Francis J, Aoki KR (2004). Plasma membrane localization signals in the light chain of botulinum neurotoxin. Proc Natl Acad Sci USA.

[b16] Ferrari E, Maywood ES, Restani L, Caleo M, Pirazzini M, Rossetto O, Hastings MH, Niranjan D, Schiavo G, Davletov B (2011). Re-assembled botulinum neurotoxin inhibits CNS functions without systemic toxicity. Toxins (Basel).

[b17] Ferrari E, Soloviev M, Niranjan D, Arsenault J, Gu C, Vallis Y, O'Brien J, Davletov B (2012). Assembly of protein building blocks using a short synthetic peptide. Bioconjug Chem.

[b18] Foran PG, Mohammed N, Lisk GO, Nagwaney S, Lawrence GW, Johnson E, Smith L, Aoki KR, Dolly JO (2003). Evaluation of the therapeutic usefulness of botulinum neurotoxin B, C1, E, and F compared with the long lasting type A. Basis for distinct durations of inhibition of exocytosis in central neurons. J Biol Chem.

[b19] Gao X, Huang L (1995). Cationic liposome-mediated gene transfer. Gene Ther.

[b20] Geisse S, Voedisch B (2012). Transient expression technologies: Past, present, and future. Methods Mol Biol.

[b21] Graham FL, van der Eb AJ (1973). A new technique for the assay of infectivity of human adenovirus 5 DNA. Virology.

[b22] Ishiyama M, Miyazono Y, Sasamoto K, Ohkura Y, Ueno K (1997). A highly water-soluble disulfonated tetrazolium salt as a chromogenic indicator for NADH as well as cell viability. Talanta.

[b23] Jahn R, Lang T, Sudhof TC (2003). Membrane fusion. Cell.

[b24] Kim TK, Eberwine JH (2010). Mammalian cell transfection: The present and the future. Anal Bioanal Chem.

[b25] Kuo CL, Oyler G, Shoemaker CB (2010). Lipid and cationic polymer based transduction of botulinum holotoxin, or toxin protease alone, extends the target cell range and improves the efficiency of intoxication. Toxicon.

[b26] Liu W, Brock A, Chen S, Schultz PG (2007). Genetic incorporation of unnatural amino acids into proteins in mammalian cells. Nat Methods.

[b27] Liu Z, Zhang N (2012). PH-sensitive polymeric micelles for programmable drug and gene delivery. Curr Pharm Des.

[b28] Lonez C, Vandenbranden M, Ruysschaert JM (2012). Cationic lipids activate intracellular signaling pathways. Adv Drug Deliv Rev.

[b29] Lord JM, Spooner RA (2011). Ricin trafficking in plant and mammalian cells. Toxins (Basel).

[b30] Madani F, Lindberg S, Langel U, Futaki S, Graslund A (2011). Mechanisms of cellular uptake of cell-penetrating peptides. J Biophys.

[b31] Menuel S, Fontanay S, Clarot I, Duval RE, Diez L, Marsura A (2008). Synthesis and complexation ability of a novel bis- (guanidinium)-tetrakis-(beta-cyclodextrin) dendrimeric tetrapod as a potential gene delivery (DNA and siRNA) system. Study of cellular siRNA transfection. Bioconjug Chem.

[b32] Montagne M, Beaudoin N, Fortin D, Lavoie CL, Klinck R, Lavigne P (2012). The Max b-HLH-LZ can transduce into cells and inhibit c-Myc transcriptional activities. PLoS ONE.

[b33] Montal M (2010). Botulinum neurotoxin: A marvel of protein design. Annu Rev Biochem.

[b34] Neumann E, Schaefer-Ridder M, Wang Y, Hofschneider PH (1982). Gene transfer into mouse lyoma cells by electroporation in high electric fields. EMBO J.

[b35] Nguyen J, Szoka FC (2012). Nucleic acid delivery: The missing pieces of the puzzle. Acc Chem Res.

[b36] Oba M, Tanaka M (2012). Intracellular internalization mechanism of protein transfection reagents. Biol Pharm Bull.

[b37] O'Brien JA, Lummis SC (2011). Nano-biolistics: A method of biolistic transfection of cells and tissues using a gene gun with novel nanometer-sized projectiles. BMC Biotechnol.

[b38] Ramos-Vara JA (2005). Technical aspects of immunohistochemistry. Vet Pathol.

[b39] Song Y, Hahn T, Thompson IP, Mason TJ, Preston GM, Li G, Paniwnyk L, Huang WE (2007). Ultrasound-mediated DNA transfer for bacteria. Nucleic Acids Res.

[b40] Tarahovsky YS, Koynova R, MacDonald RC (2004). DNA release from lipoplexes by anionic lipids: Correlation with lipid mesomorphism, interfacial curvature, and membrane fusion. Biophys J.

[b41] Tros de Ilarduya C, Sun Y, Duzgunes N (2010). Gene delivery by lipoplexes and polyplexes. Eur J Pharm Sci.

[b42] Wang J, Zurawski TH, Meng J, Lawrence G, Olango WM, Finn DP, Wheeler L, Dolly JO (2011). A dileucine in the protease of botulinum toxin A underlies its long-lived neuroparalysis: Transfer of longevity to a novel potential therapeutic. J Biol Chem.

[b43] Wong AW, Scales SJ, Reilly DE (2007). DNA internalized via caveolae requires microtubule-dependent, Rab7-independent transport to the late endocytic pathway for delivery to the nucleus. J Biol Chem.

[b44] Zelphati O, Wang Y, Kitada S, Reed JC, Felgner PL, Corbeil J (2001). Intracellular delivery of proteins with a new lipid-mediated delivery system. J Biol Chem.

