# Supplementary material for: Unexpected Transcellular Protein Crossover Occurs During Canonical DNA Transfection
Source: J Cell Biochem. 2014 Oct 15;115(12):2047–54. doi: 10.1002/jcb.24884 (PMC4263260; doi:10.1002/jcb.24884)
Supplement: Supplementary file 1 — Supporting Information. [file jcb0115-2047-SD1.doc]

Supplementary figure 1: Western immunoblotting of sampled medium and resuspended cells. The medium taken from EGFP-LcA transfected Neuro2A cells did not contain any detectable levels of SNAP25 while resuspended cells display a near complete SNAP25 cleavage.

Supplementary information

**Materials and method**

**Cell culture**

Mouse Neuro2A cells (ATCC: CCL-131; LGC Standards; Teddington, UK) were grown in a 37^o^C incubator at 5% CO_2_, fed with low glucose Dulbecco’s Modified Eagle Medium (DMEM; Gibco; Paisly, UK) supplemented with 10% Fetalclone1 Calf Serum (FCS; HyClone; Fisher Scientific; Loughborough, UK) and 1% penicillin/streptomycin (P/S) (Invitrogen; Paisley, UK). Every 3-4 days, cells were washed with Phosphate buffered saline (140 mM NaCl, 2.5 mM KCl, 19 mM Na_2_PO_4_, 2 mM KH_2_PO_4_; PBS), resuspended in culture medium using flow pressure, and counted by hemacytometer. Cells were plated at 1 x 10^6^ cells per 9 cm culture dish (BD Biosciences; San Jose, USA), at 8 x 10^4^ cells per well in uncoated 24 well plates (BD Biosciences) with or without cover slips, or at 8 x 10^3^ cells per well in uncoated 96 well plates (BD Bioscience).

**Protein, Peptide and DNA synthesis**

BoNT/A1 holoenzyme (LcA) was prepared as previously described . Protein concentration was determined by Pierce BCA protein assay kit (Thermo-scientific; Loughborough, UK) according to manufacturer protocol. EGFP-LcA and EGFP-LcE had been inserted into pcDNA3.1 as previously described . The ricin A-chain from *Ricinus Communis* was purchased from Sigma-Aldrich (Dorset, UK). FITC-Syntaxin (201-245) was synthesized by Peptide Synthetics (Southampton, UK) and was previously described elsewhere .

**Transfection and transduction**

Transfection was performed 24 h after plating. The DNA and transfection reagent were pre-incubated for 30 min in 100 µl of Opti-MEM (GIBCO) with 2.5 µl of transfection reagents per 500 µl of culture medium in a 24 well plate (or with 400 µl of Opti-MEM with 10 µl of transfection reagents per 10 ml of culture medium in 9 cm petri dishes). Lipofectamine (Invitrogen), Lipofectamine 2000, Lipofectamine LTX (Invitrogen), and Fugene HD (Promega; Southampton, UK) were used as received from manufacturer. Cells were incubated with proteins or transfection reagents at 37^o^C in cell culture incubator for 18 h, washed with PBS, the medium was replace, and cell were left for 42 h or as otherwise indicated. Sampled medium was gently taken directly from the petri dishes or wells at the indicated time points and maintained at 4^o^C until needed and were kept for a maximum of 14 days. 200 µl of this medium was added to 400 µl of pre-plated Neuro2A cells in 24 well culture dishes. The sampled medium, when indicated, was also centrifuged at 20 000 rcf for 10 min to separate soluble and suspended fractions. Supernatant was added as described while precipitated pellet was subsequently resuspended in the same volume of culture medium before being added onto pre-plated Neuro2A cells. Peptides were added to LTX treated or untreated Neuro2A at 10 µg/ml and incubated for 30 min at 37^o^C as described elsewhere .

**Confocal miscroscopy**

Cells grown on cover slips in 24 well plates were washed once with PBS, and were fixed by incubated in 4% Paraformaldehyde (PFA; Alfa Aesar; Haysham, UK) in PBS for 20 min at room temperature (RT). Wells were washed 3 times with PBS for 5 min and incubated 10 min in 10 mM NH_4_Cl (Sigma-Aldrich). The cells were then incubated for 30 min in a permeabilization solution composed of 0.1% Triton X-100 (Sigma-Aldrich) and 5% Bovine serum albumin (BSA; Sigma-Aldrich) in PBS at RT. Permeabilization solution was removed and replaced with 5% BSA in PBS containing rabbit polyclonal anti-cleaved SNAP25 antibody diluted to 1:500 for 90 min at RT. The wells were washed 3 times in PBS, then incubated for 30 min with Alexa Fluor® 594 goat anti-rabbit IgG (H+L) (Invitrogen) diluted 1:800 in 5% BSA in PBS at RT. Cells were counterstained with Hoechst 33342 (Fisher) at 1:10 000 dilution in PBS for 5 min. Wells were washed 3 times in PBS. Cover slips were overturned onto Vectashield (Vectorlabs; Orton Southgate, UK) mounting medium, and sealed with nail polish. For live imaging, cells were counterstained with Hoechst 33342 directly added to the medium, washed, then visualized through glass bottom culture dishes (MAT laboratories). Lysotracker green DND-26 (Life technology, Paisley, UK) was used for live cell imaging following manufacturer's protocol. Briefly, a final dilution of 1:20 000 was added directly to the cell culture solution and incubated for 30 min at 37^o^C. Slides and dishes were visualized on Zeiss 710 (Cambridge, UK) on 10 or 63X. The fluorescent gains intensities and pinhole size (1 AU) were identical between experimental samples.

**Western immunoblotting**

Immediately after the medium was removed, cells were incubated for 5 min in 100 µl loading buffer (56 mM SDS (Sigma-Aldrich), 0.05 M Tris-HCl (BioRad; Hemel Hempstead, UK) pH 6.8, 1.6 mM UltraPure EDTA (Gibco), 6.25% glycerol (Fisher Scientific), and 0.00001% bromophenol blue (Fisher Scientific) per 24 well culture dish. One unit of benzonase (Novagen; EMD Millipore) supplemented with 1 µl of 1 M MgCl_2_, was added to each well and plates were shaken at 1500 rpm for an additional 10 min. Samples were boiled for 1 min at 95^o^C then run on 12% Bis-Tris SDS-PAGE gels (Invitrogen). Migrated samples were transferred on Immobilin-P Polyvinylidene fluoride (PVDF) membranes (EMD Millipore), and then incubated for 30 min in blotting solution (5% milk, 0.1% TWEEN 20 (Thermo Scientific) in PBS). Mouse monoclonal anti-SNAP25 (SMI81; Novagen; EMD Millipore; Feltham, UK) diluted to 1:3000 was incubated for 1 h at RT in blotting solution. Membranes were washed 3 times in 0.1% TWEEN 20 in PBS for 5 min and then incubated for 30 min in blotting solution with secondary stabilized peroxidase-conjugated goat anti-mouse (H+L) at RT. Membranes were washed 3 times for 5 min in 0.1% TWEEN 20 in PBS. Bands were illuminated using SuperSignal West Dura Extended Duration Substrate (Thermo Scientific) and signal was visualized by autoradiogram using Fuji Medical X-Ray films (Ross-on-wye, UK). PVDF membranes were subsequently stained with Coomassie (Fisher Scientific).

**Cell proliferation assays**

Cells plated into 96-well plates (Costar; Sigma-Aldrich), exposed to 1/10 of the amount of the transfection recipe indicated above, were incubated for 40 h with indicated compounds. Cell Counting Kit-8 (CCK-8; Sigma-Aldrich) was used to determine cell survival . Assay was performed according to manufacturers protocol. A Tecan Safire^2^ microplate reader (Männerdorf, Switzerland) was used to read absorbance at 450 nm in each well.

**Flow cytometry**

Treated cells were washed twice with PBS centrifuged at 300 g for 5 minutes and resuspended in 10 mM HEPES (Fisher Scientific), 140 mM NaCl, 2.5 mM CaCl_2_, pH 7.4 into 12 x 75 mm round bottom test tube (Scientific Laboratory Supplies; Wilford, UK). Fluorescent intensities of the cell populations were measured using FACScalibur 2 (BD Bioscience). For cell survival experiments a propidium iodide solution (P-3566; Life technology) diluted to a final concentration of 2 µg/ml and an Annexin V FITC solution (A9210; Sigma-Aldrich) diluted to a final concentration of 100 µM were added to the cells and incubated in the dark at room temperature for 10 minutes. Cells were centrifuged at 300 g for 5 minutes then resuspended gently in HEPES buffer. Proper gating and laser intensity were determined with non-transfected or non stained controls respectively. Histographic distribution containing 99% of control cells was used as a threshold for the EGFP signal. Untreated cells were used to gate the propidium iodide and Annexin V-FITC signals respectively. Data was analyzed using FlowJo version 9.4.4 (Tree Star Inc.; Ashland, USA).

**Statistical Analysis**

All experiments were performed in at least three independent experiments. Results are presented as mean ± standard deviation (SD). Data analysis was performed using Graphpad Prism 5.0 (La Jolla, USA). A p<0.05 was considered statistically significant.
