# Supplementary figures and images for: Unexpected Transcellular Protein Crossover Occurs During Canonical DNA Transfection
Source: J Cell Biochem. 2014 Oct 15;115(12):2047–54. doi: 10.1002/jcb.24884 (PMC4263260; doi:10.1002/jcb.24884)

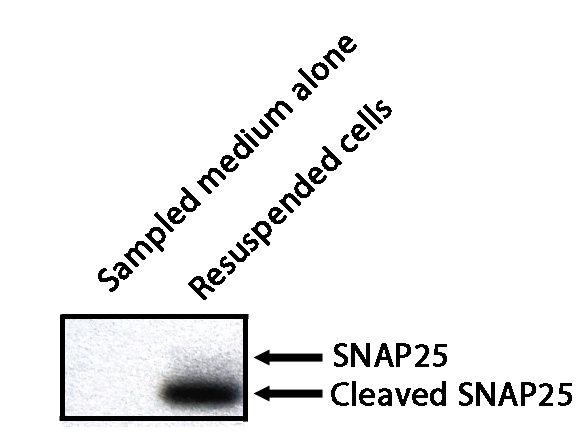

Supplement: Supplementary file 2 — Supporting Information. [file jcb0115-2047-SD2.tif]
